# Separation of the Biofuel Methyl Ethyl Ketone from Aqueous Solutions Using Avocado-Based Activated Carbons: Synthesis Conditions and Multilayer Adsorption Properties

**DOI:** 10.3390/molecules30163426

**Published:** 2025-08-20

**Authors:** Hilda Elizabeth Reynel-Avila, Eduardo Ledea-Figueredo, Lizbeth Liliana Díaz-Muñoz, Adrián Bonilla-Petriciolet, Ismael Alejandro Aguayo-Villarreal, Laura Gabriela Elvir-Padilla, Carlos Javier Durán-Valle

**Affiliations:** 1 Departamento de Ingeniería Química, Tecnológico Nacional de México—Instituto Tecnológico de Aguascalientes, Aguascalientes 20255, Mexico; eduardoledea@gmail.com (E.L.-F.); lizbeth_liliana_3@hotmail.com (L.L.D.-M.); petriciolet@hotmail.com (A.B.-P.); 2 SECIHTI Investigadoras e Investigadores por México, Ciudad de México 03940, Mexico; 3 Facultad de Ciencias Químicas, Universidad de Colima, Comila 28040, Mexico; 4 Unidad Académica Multidisciplinaria Reynosa, Universidad Autónoma de Tamaulipas, Tamaulipas 88740, Mexico; laura_gabriela_10@hotmail.com; 5 Instituto del Agua, Cambio Climático y Sostenibilidad, Universidad de Extremadura, 06006 Badajoz, Spain

**Keywords:** biorefineries, avocado seed, carbonaceous adsorbents, downstream separation, methyl ethyl ketone

## Abstract

This study reports the separation of methyl ethyl ketone (MEK), a relevant compound in the biorefinery context, from aqueous solutions using activated carbons derived from avocado seed biomass. Two synthesis routes were explored via chemical and thermal activation with H_2_SO_4_ and KOH. A Taguchi experimental design was applied to tailor synthesis conditions, with MEK adsorption capacity as the target property. Adsorption kinetics and isotherms were evaluated to determine the thermodynamic behavior of MEK separation using the best-performing activated carbons. The carbon activated with H_2_SO_4_ achieved the highest adsorption capacity (142 mg g^−1^) at 20 °C and pH 4, surpassing KOH-based materials. This enhanced performance correlated to increased surface area and acidic oxygenated functionalities. However, higher pH and temperature reduced the adsorption efficiency for all adsorbents. Comprehensive characterization was performed using XRD, XRF, FTIR, SEM, N_2_ adsorption–desorption isotherms, pH at point of zero charge, and surface acidity/basicity analysis via Boehm titration. Thermodynamic data and surface characterization indicated that MEK adsorption occurs via a double-layer mechanism dominated by electrostatic interactions and hydrogen bonding. The findings highlight an optimized approach for tailoring avocado-based activated carbons to efficiently recover MEK from aqueous media, supporting its potential application in downstream purification of fermentation broths for biofuel production and energy transition processes.

## 1. Introduction

The U.S. Department of Energy has indicated that ketone-based chemicals are among the most promising fuel families that can be incorporated to diversify the global energy mix [1]. It has been suggested that spark-ignition engines can operate using methyl ethyl ketone (MEK) as a potential next-generation biofuel [1,2]. MEK is also an important industrial commodity that can be used as an organic solvent for coatings, paintings, printing inks, pharmaceuticals, and other products [3,4]. Lee et al. [5] have analyzed the MEK market, reporting a global demand of 1.42 million tons with an expected projection of 2.11 million tons and 4.01 billion dollars for 2024.

MEK is a compound derived mainly from petroleum; however, it can also be produced in biorefineries via the pyrolysis or sugar fermentation of lignocellulosic biomass [2,6]. It is also noteworthy that different manufacturing industries discharge residual effluents polluted by this chemical. Consequently, its separation and recovery are paramount as a value-added product that can be used in energy transition, as well as in terms of mitigating the environmental impact of its supply chain via the reduction of emissions to air and soil, as MEK is a toxic compound [3,4]. Different methods have been used for separating MEK from aqueous matrices, including distillation, adsorption, air stripping, and biofiltration [3,7,8]. Combustion, ozonation, and catalysis can be applied if MEK degradation is the primary goal [8].

Activated carbon-based separation is an effective and reliable method for recovering value-added compounds from fluids and removing contaminants. This process has several applications in industries where the adsorbent selection and identification of appropriate operating conditions contribute to improving the cost-effectiveness tradeoff [9,10]. Studies reporting MEK recovery via adsorption have mainly focused on gaseous phase separation because it is a volatile organic compound under moderate operating conditions (i.e., its normal boiling point is 79.64 °C) [3,11,12,13,14]. In contrast, MEK adsorption from water has not been widely investigated, even though its production from fermentation processes offers techno-economic advantages [8,15,16,17]. For instance, Sotelo et al. [15] and Uguina et al. [16] applied granulated activated carbon (GAC-1240) and agglomerated silicalite for MEK recovery from aqueous solutions in batch and continuous operating configurations. GAC-1240 achieved a maximum MEK adsorption capacity of 60 mg g^−1^ at 25 °C, whereas that of silicalite was 69 mg g^−1^. This activated carbon was regenerated via a thermal route using nitrogen or air (as a purging gas), where it was observed that its MEK adsorption properties were reduced during the desorption/adsorption cycles. In contrast, the regenerated silicalite did not exhibit a significant decrease in its adsorption capacity. Ali and Mohammed [17] recovered MEK from aqueous solutions using siliceous rocks. This material was composed of metallic oxides (e.g., Si, Ca, Mg, and Al) with a maximum MEK adsorption capacity of 470 mg g^−1^ at 25 °C and pH 7. MEK separation was exothermic, and the decrease in the solution pH favored its adsorption because the adsorbent surface was negatively charged in acidic media, leading to higher electrostatic attraction with MEK molecules. The separation of MEK from binary solutions containing diethyl ketone (DEK) using a fixed-bed column packed with sepiolite has also been studied [8]. The maximum MEK adsorption capacity calculated using the Yoon–Nelson and dose–response models was 15 mg g^−1^. These results were used to propose a bio-barrier that combines sepiolite and Streptococcus biofilm for the pilot-scale treatment of industrial wastewater containing DEK, MEK, and heavy metals.

The production cost of commercial adsorbents and their limited separation performance, due to the lack of tailored synthesis routes, can affect the application of adsorption-based equipment to recover MEK from fermentation broths. The use of residual materials as adsorbent precursors is an attractive alternative, from both environmental and economic perspectives, to improve the operation of biorefineries that produce MEK and other value-added products [9,10,17,18]. Different sources of residual lignocellulosic biomass have been assessed to obtain carbon-based adsorbents because of their renewable characteristics and physicochemical properties, such as surface chemistry, reactivity, and low density [19,20]. This type of feedstock allows a relatively low-cost and straightforward conversion to carbon-based materials, which can achieve competitive separation for recovering compounds from aqueous matrices [21,22,23]. Examples of lignocellulosic residues that can be used to prepare carbon-based adsorbents include peach seeds [24], wheat straw [25], avocado seeds [26,27], almond shells [28], and coffee residues [29]. Avocado seeds are widely available in Mexico, making them attractive precursors for the preparation of activated carbon for local industrial consumption. This residual biomass has been valorized to prepare new materials for the separation of ammonium [26], fluoride [27], dyes [30], heavy metals such as chromium [31] and cadmium [32], phenolic compounds [33], arsenic [34], pharmaceutical products [35], and other adsorbates from water. However, to the best of the authors’ knowledge, activated carbons obtained from avocado seeds have not been applied in MEK recovery from aqueous solutions. This context contrasts with previous studies, which have focused primarily on MEK separation in the gas phase using commercial materials or adsorbents derived from another residual biomass [36,37].

The adsorption properties of a material are determined by its pore structure and surface chemistry. Surface functionalization can alter the selectivity of adsorbents, thereby influencing their adsorption capacities for separating specific organic molecules [11]. The adsorbent properties can be tailored by manipulating the synthesis and activation routes using different methodologies, including chemical oxidation, wet impregnation, microwave heating, and plasma treatment [38]. Chemical oxidation involves the incorporation of oxygenated functionalities on the adsorbent surface and offers techno-economic benefits compared to other activation strategies [39]. The introduction of oxygen-containing functionalities modifies the polarity of adsorbents and can improve their separation properties for polar organic molecules, such as MEK [11,38]. Acidic and basic solutions can be utilized for the chemical activation of adsorbent surfaces. Some activators include phosphoric and sulfuric acids, potassium and sodium hydroxides, calcium, and zinc chlorides [40,41,42,43]. The effectiveness of surface modification depends significantly on the identification of the best chemical activation conditions, which is usually a multivariable problem that should be resolved by applying proper experimental designs and statistical tools. 

Therefore, this study highlights the valorization of avocado seeds, which are an abundant and inexpensive residue, to produce carbon-based adsorbents for the separation of MEK from aqueous solutions. The tailoring of the surface properties of avocado-based carbonaceous adsorbents for MEK recovery was performed, including a detailed analysis of the impact of their preparation conditions. An integrated approach based on Taguchi experimental methodology, adsorbent surface characterization, and mechanistic modeling was implemented. Kinetic and equilibrium studies to separate MEK from aqueous solutions were quantified and modelled, under various operating scenarios, using the best activated carbon. An explanation for the MEK adsorption mechanism was also proposed, applying the surface characterization results, density functional theory (DFT) simulations, and statistical physics theory. The main findings reported in this manuscript will help to improve the recovery and purification of MEK from fermentation broths in the operational context of biorefineries, which is a promising chemical for energy transition and a relevant industrial feedstock. 

## 2. Results and Discussion

### 2.1. Identification of the Best Avocado-Based Activated Carbon for MEK Separation

The pyrolysis yields for obtaining the avocado-based chars and their values of pH at the point of zero charge (pH_pzc_) for the tested preparation conditions are reported in Table 1. The pyrolysis yields ranged from 22 to 26% and decreased as the temperature and dwell time increased. This trend was mainly due to the degradation and volatilization of organic and inorganic matter contained in the residual biomass, where more drastic pyrolysis conditions reduced char yields. These values agreed with those reported by other authors for the pyrolysis of this lignocellulosic residue, with yields of 21–29% [44], 20–25% [45], 20–21% [46], and 12–25% [27]. The pH_pzc_ values of the avocado-based chars were in the range of 6.75–7.19, where a slight increase was observed as the dwell time and pyrolysis temperature increased. This result could be related to the loss of acidic oxygen-containing groups (e.g., carboxylic, phenolic, and lactonic groups) caused by the application of harsh conditions for biomass thermochemical conversion. This loss of functional groups generated an increase in the surface basicity of the avocado chars, which was reflected by an increase in their pH_pzc_ [47]. Comparable pH_pzc_ values have been reported for pyrolyzed materials obtained from avocado seeds (6.40), hazelnut shells (7.3), and wood from beer barrels (6.8) [48,49].

**Figure 1:**
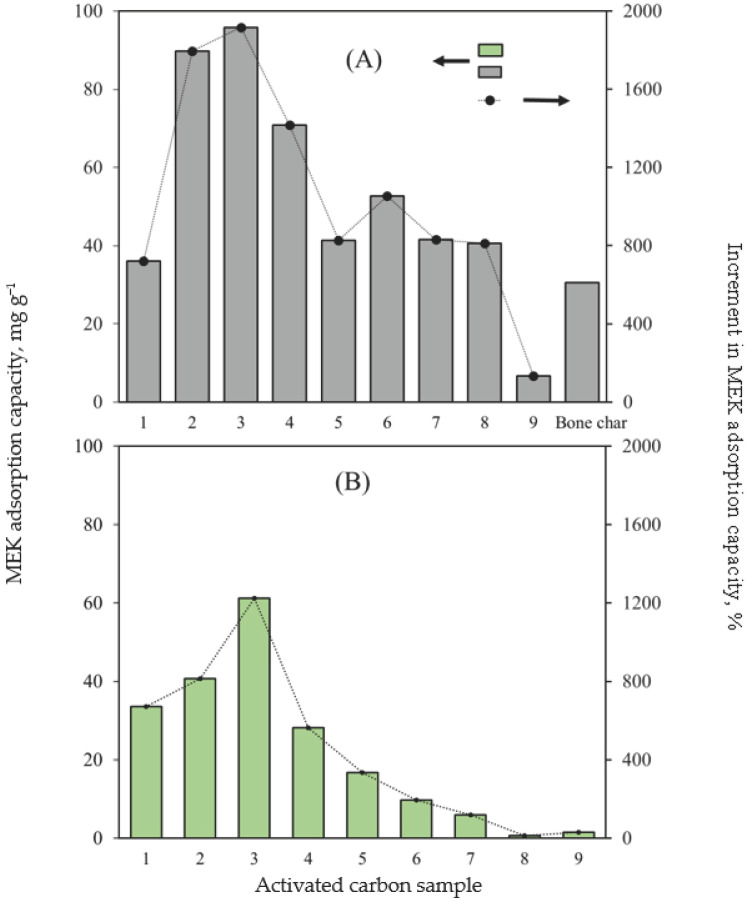
MEK adsorption capacities of activated carbons prepared with H_2_SO_4_ (**A**) and KOH (**B**) using a Taguchi L_9_ design.Overall, the activated carbon samples No. 3A (H_2_SO_4_) and 3B (KOH) displayed the highest MEK adsorption capacities with values of 100.8 and 66.2 mg g^−1^, respectively. Both samples were pyrolyzed at 700 °C for 6 h, activated with a 1 M solution (either H_2_SO_4_ or KOH), and thermally treated at 900 °C for 2 h. These avocado-based activated carbon outperformed the MEK adsorption capacity of the tested commercial bone char (30.5 mg g^−1^). Herein, it is convenient to indicate that the preparation of activated carbon using residues of avocado seeds offers advantages in terms of cost and sustainability. As indicated, avocado seeds are an abundant agro-industrial residue with a relatively low cost, which can help to reduce the production overhead compared to conventional adsorbents derived from high-purity precursors. The thermochemical transformation of this residue into functional materials aligns with the circular economy by valorizing waste and residues, and minimizing the environmental impact associated with landfill disposal or incineration. Consequently, these biowaste-based adsorbents represent a competitive and sustainable alternative for MEK separation.

MEK adsorption capacities of 18 activated carbon samples and their increment (%) compared to the separation performance of avocado chars, using H_2_SO_4_ (samples labeled as A) and KOH (samples labeled as B) as chemical activators, are shown in Figure 1. MEK adsorption capacity of avocado-based chars was <5 mg g^−1^, while the commercial bone char achieved a MEK separation of 30.5 mg g^−1^ under the tested experimental conditions.

Avocado-based adsorbents activated with H_2_SO_4_ and KOH showed increments in their MEK adsorption capacities of up to 1917% and 1224%, respectively, considering the separation performance of avocado chars without surface functionalization. These results confirm the effectiveness of the proposed activation routes for improving the MEK adsorption properties of avocado-based activated carbons. The adsorbents prepared with H_2_SO_4_ exhibited higher MEK adsorption capacities than those activated with KOH did. MEK adsorption capacities of activated carbons prepared with H_2_SO_4_ ranged from 11.6 to 100.8 mg g^−1^, whereas those synthesized with KOH displayed adsorption capacities of 5.7–66.2 mg g^−1^. The results confirmed the superior performance of acidic adsorbents compared with that of alkaline materials for MEK separation from aqueous solutions. Similar results have been reported in other studies on the separation of different adsorbates using activated carbons prepared from biomass [50,51]. For instance, Ogungbenro et al. [50] prepared adsorbents from date seeds using H_2_SO_4_ and KOH as chemical activators, and they achieved CO_2_ adsorption capacities of 78.7 and 16.9 mg g^−1^, respectively. Zhou et al. [51] prepared a char from wheat straw modified with KOH, HCl, or HF for phenol adsorption. The results indicated a clear trend in the phenol adsorption capacities of adsorbents prepared with different activators: HF > HCl > NaOH. Although the cited studies focused on different adsorption systems, they highlight the benefits of chemical activators to tailor surface properties and pore structures of carbon-based materials.

**Figure 2:**
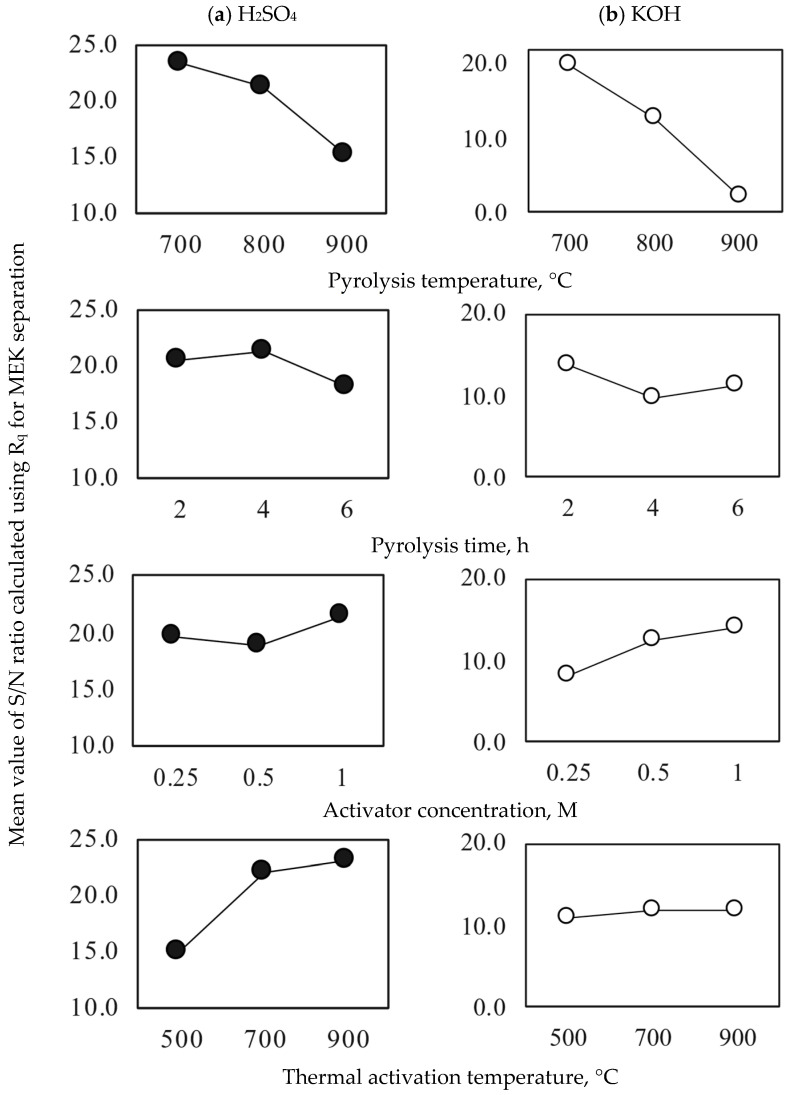
Signal-to-Noise ratio analysis for the preparation of avocado-based activated carbons used in MEK adsorption from aqueous solutions. Activators: (●) H_2_SO_4_ and (○) KOH.

The impact of avocado-based activated carbon preparation conditions on MEK separation in terms of the S/N ratio is shown in Figure 2. For the two chemical activators, it was observed that increasing the avocado biomass pyrolysis conditions reduced the S/N ratio (i.e., MEK separation). More severe pyrolysis conditions of avocado biomass affected the concentration of oxygenated functional groups on the adsorbent surface, although the textural parameters improved. This result agreed with the findings reported by Qurat-ul-Ain et al. [52], who synthesized activated carbon using *Parthenium hysterophorus* as a precursor. They observed that as the pyrolysis temperature increased, the oxygen content on the carbon surface decreased. This phenomenon was attributed to the decomposition of oxygenated bonds and the release of low-molecular-weight byproducts. In contrast, the MEK adsorption properties of the activated carbon samples improved with increasing activator concentration and thermal activation temperature. Both variables had a direct effect on the functionalization of carbonaceous adsorbent surfaces [53]. Similar results have been reported for the preparation of activated carbon for various separation tasks [54,55]. Specifically, Zakaria et al. [54] assessed the effects of H_3_PO_4_ ratio (3, 4, and 5) and pyrolysis temperature (300, 400, and 500 °C) on the adsorption capacity of mangrove-based adsorbents for methylene blue removal. The results showed that increasing the H_3_PO_4_ ratio from 3 to 4 improved the adsorption capacity from 67.1 to 72.3 mg g^−1^; however, a further increase to a ratio of 5 generated an adsorbent with a lower adsorption capacity. They also observed a reduction in the adsorbent performance as the activation temperature increased, particularly in combination with higher H_3_PO_4_ ratios. These authors also found a direct correlation between these factors and the adsorbent surface area—that is, as the surface area increased, methylene blue removal improved. This study concluded that the synthesis route is a key factor that influences adsorbent separation performance. Tetteh et al. [55] analyzed the effect of preparation conditions on the adsorption properties to separate different adsorbates, such as heavy metals and dyes. They also observed that the type of activation agent (physical or chemical) directly affects the adsorption capacity. 

The ANOVA results reported in Table S1 indicate that the temperatures of avocado seed pyrolysis and thermal activation were the main variables with the highest influence on the MEK adsorption properties of activated carbons prepared with H_2_SO_4_. Specifically, the impact of the preparation conditions on the MEK separation using H_2_SO_4_-based activated carbons followed the order: pyrolysis temperature > thermal activation temperature >> pyrolysis time > H_2_SO_4_ concentration. For the experimental design using KOH, the variable with the highest influence on the MEK adsorption properties of activated carbons was also the pyrolysis temperature, as shown in Figure 2 and Table S1.

The statistical relevance of KOH-based preparation conditions was as follows: pyrolysis temperature >> KOH concentration > pyrolysis time >> thermal activation temperature. These trends agreed with the findings of other studies on activated carbon preparation, in which more functional groups were developed on the char surface at lower pyrolysis temperatures [56]. The application of high pyrolysis temperatures causes the degradation and loss of structural functionalities that can participate in MEK separation [57]. However, the content of functional groups can increase with the thermal activation and chemical functionalization of avocado char, which contributes in two ways: the transformation of internal groups from the adsorbent structure and the development of new active sites [39]. These findings were consistent with the results of the activated carbon characterization. Specifically, activated carbons obtained with H_2_SO_4_ presented pH_pzc_ values of 1.97–2.72, while the adsorbents prepared with KOH showed pH_pzc_ values of 11.23–12.86. The concentration of acidic sites of the best activated carbon samples (which was determined by Boehm titration) was 1.73 mmol g^−^^1^ (3A) and 0.48 mmol g^−1^ (3B), while char No. 3 showed a concentration of acidic sites of 0.71 mmol g^−^^1^. The results also indicated that carboxylic sites prevailed on the surface of the tested samples. The basic site concentration of these adsorbents was: 0.42 mmol g^−^^1^ (char No. 3) < 0.46 mmol g^−^^1^ (activated carbon 3A) < 1.14 mmol g^−^^1^ (activated carbon 3B). The acidic surface character of the activated carbon sample 3A favored MEK separation. The pH_pzc_ values of these adsorbents were 2.72, 6.89, and 11.23 for 3A, char No. 3, and 3B, respectively. It is convenient to recall that the acidic sites include carboxylic, phenol, and hydroxyl groups [39], where the development of hydroxyl and carboxylic functionalities increases the carbon surface polarity [58], favoring the adsorption of polar organic molecules. Carbonyl, chromene, pyrone, and ether functionalities are commonly associated with the basic properties of carbonaceous materials [59]. The low pH_pzc_ value of sample 3A is evidence of the higher abundance of acidic surface groups.

**Figure 3:**
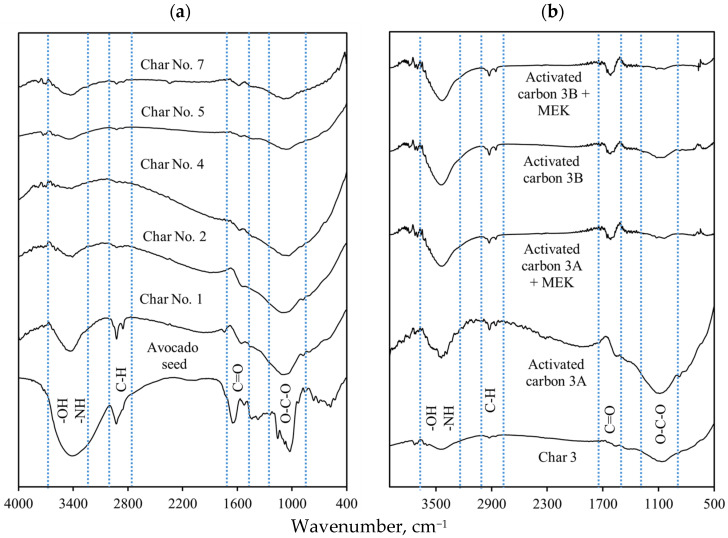
FTIR spectra of avocado-based activated carbons used in MEK separation from aqueous solutions. (**a**) Avocado seed char, (**b**) Avocado seed activated carbon.

FTIR spectra of the avocado seed biomass, char samples, and activated carbons 3A and 3B, before and after MEK separation, are reported in Figure 3. The main functional groups associated with the composition of avocado seed (lignocellulose) were identified in the tested samples. Specifically, OH (carboxylic and phenolic) and NH groups were identified with the absorption bands at 3700–3300 cm^−^^1^ [44,60,61,62], while CH groups were associated with the absorption bands located in the 2950–2800 cm^−^^1^ region [31,63]. The absorption bands at 1750–1620, 1550, and 1300–1000 cm^−^^1^ corresponded to C=O, COO, and C–O stretching vibrations of carbonyl and carboxylic groups [64,65]. The lignin aromatic structures (C=C) were identified by the absorption band at 1500 cm^−^^1^ [66,67], and the C–O and C–O–C groups (from acids, alcohols, phenols, and esters) were located with the absorption bands at 1260–1000 cm^−^^1^ [68,69]. The absorption bands at 885, 840, and 775 cm^−^^1^ corresponded to the C–H out-of-plane deformation vibrations of the benzene rings [70,71]. The out-of-plane OH bending group was observed with the absorption band at 620 cm^−^^1^ [68,72]. It was found that an increase in pyrolysis temperature and dwell time generated changes in the intensities of the absorption bands of the main organic functional groups of carbonaceous adsorbents.

The intensities of the absorption bands located at 3400–3300 (OH), 1750–1620 (C=O, COO, C–O), and 1260–1000 cm^−^^1^ (C–O and C–O–C) decreased, which may be related to condensation reactions and/or lignin cleavage, decarboxylation, as well as with the partial removal of cellulose and hemicellulose during the adsorbent preparation [73,74,75]. FTIR spectrum of activated carbon 3A (H_2_SO_4_) showed evident changes from that of avocado char No. 3. The absorption bands of OH (3350 cm^−^^1^), C–O (1070 cm^−^^1^), and C=C (1500 cm^−^^1^) widened and increased in their intensities. In contrast, the spectrum of activated carbon 3B (KOH) showed changes in the absorption bands at 3350 (OH), 1750–1620 (C=O, COO), and 1300–1000 (C–O) cm^−^^1^ [31]. These changes corroborated the impact of chemical activation on adsorbent surface chemistry, where the formation of oxygenated functionalities (mainly carboxylic groups) was favored, especially for sample 3A, which was already confirmed by its acidic site concentration from Boehm titration and pH_pzc_. As indicated, the oxygen-containing functional groups increased after the char activation, where the total concentration of functional groups of activated carbon 3A was higher than that of activated carbon 3B. 

For samples loaded with MEK, it was observed that the absorption bands of the OH group at 3440 cm^−^^1^ and the C–O group at 1100 cm^−^^1^ decreased in the spectrum of activated carbon 3B, while the absorption bands at 1100–1000 cm^−^^1^ associated with C–O and C–O–C groups also decreased in the FTIR spectrum of sample 3A. These changes could be related to the participation of these functional groups in MEK adsorption via hydrogen bonding and electrostatic interactions [76]. For both activated carbon samples, the C=O absorption band at 1700 cm^−^^1^ increased slightly, indicating the incorporation of MEK molecules on the adsorbent surface during the adsorption process.

**Figure 4:**
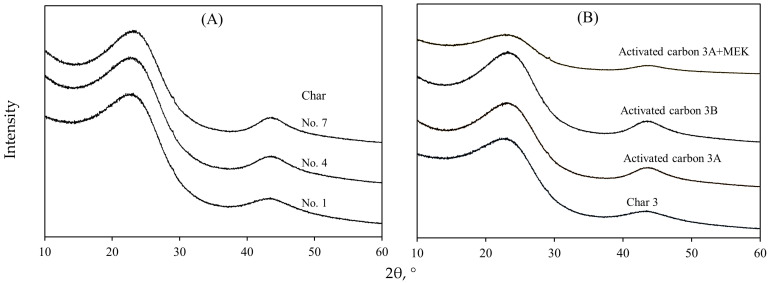
XRD analysis of avocado-based (**A**) chars and (**B**) activated carbons used in MEK separation from aqueous solutions.

Figure 4 shows the X-ray diffractograms of avocado chars obtained under different pyrolysis conditions and the best activated carbon samples used for MEK adsorption. All the patterns presented two broad peaks at ~23 and ~43 °2θ, which indicated a graphitic structure with an amorphous nature and low crystallinity [29,77]. The diffraction patterns of avocado chars were similar, suggesting that the pyrolysis conditions did not significantly affect their graphitic and crystalline structures. A variation in the crystallinity of the activated carbon samples after MEK adsorption was observed, which agreed with the results reported in other studies [78,79].

XRF results are reported in Table 2. The biomass precursor contained K (3% by mass), Ca (1.2% by mass), P (0.4% by mass), and Mg (0.12% by mass), while these elements were concentrated in the char and activated carbon samples. BET surface areas of char No. 3 and activated carbons 3A and 3B were 265, 421, and 309 m^2^ g^−1^, respectively. Total pore volumes of these samples were 0.16, 0.23, and 0.17 cm^3^ g^−1^, while their average pore diameters were 18.6, 17.6, and 16.6 Å. According to the IUPAC classification, these pore diameters confirm that the porosity of these materials is predominantly microporous. These results showed that H_2_SO_4_, KOH, and the thermal activation of avocado chars improved the adsorbent surface area up to 59% and 17% for samples 3A and 3B, respectively.

**Figure 5:**
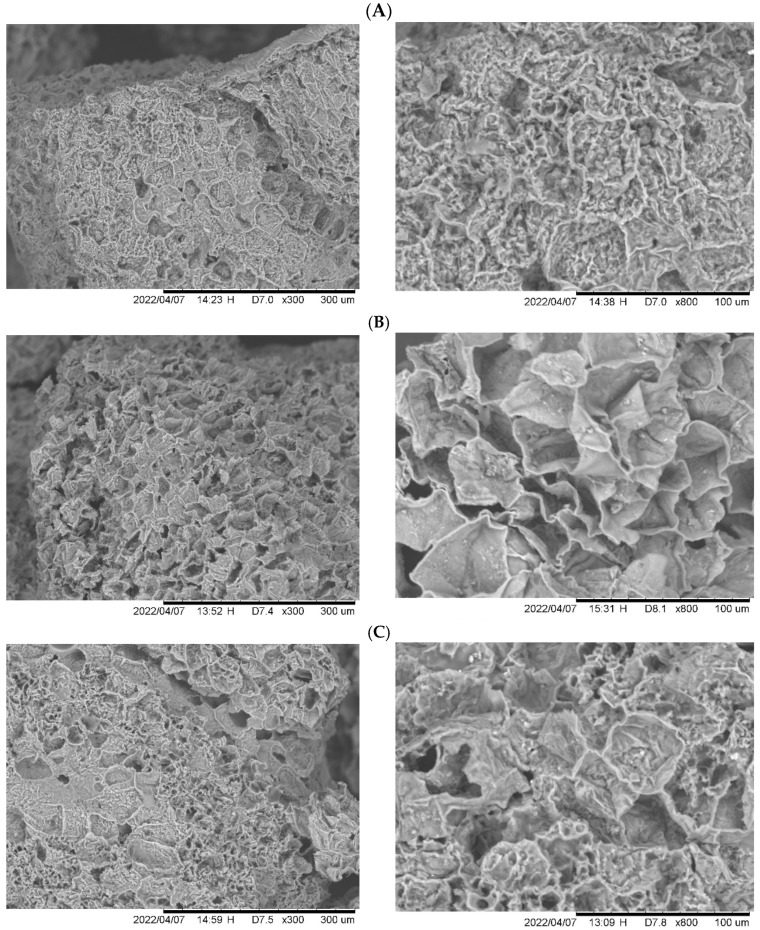
SEM micrographs of (**A**) char 3, (**B**) activated carbon 3A, and (**C**) activated carbon 3B.

SEM micrographs showed that chars had a rough and compact surface with fewer pores or channels than those of activated carbon samples, especially adsorbent 3A, see Figure 5. H_2_SO_4_ activation generated a type of fiber with irregular sheet shapes. It was also observed that KOH activation did not significantly modify the surface morphology as H_2_SO_4_ did, where the structure of activated carbon 3B was compact with channels.

### 2.2. Thermodynamics of MEK Separation Using Avocado-Based Activated Carbons

**Figure 6:**
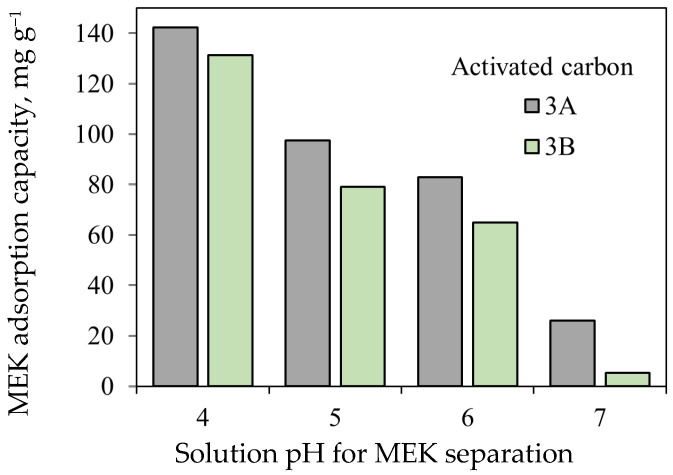
MEK adsorption capacities of activated carbons 3A and 3B at different pH values and 20 °C.

Activated carbon samples 3A and 3B were used to analyze the thermodynamics of MEK separation. The effect of aqueous solution pH on MEK adsorption using these adsorbents is reported in Figure 6. The highest MEK adsorption capacities were obtained at pH 4 with values of 135 and 118 mg g^−1^ for samples 3A and 3B, respectively. At pH 7, the adsorption capacities of both samples decreased significantly (up to 96%), reaching values of 26 mg g^−1^ for 3A and 5 mg g^−1^ for 3B, respectively.

Overall, it was observed that MEK separation increased with increasing acidity of the aqueous solutions. However, MEK remains a neutral molecule at tested pH conditions because its pKa = 14.7 [80]. This trend is explained by the surface charge and chemical functionalities of the activated carbon samples. The surface of the adsorbent is positively charged when the solution pH < pH_pzc_ (e.g., pH 4 < pH_pzc_ = 11.23 for sample 3B). This condition enhances electrostatic interactions with the polar MEK molecules, in addition to the presence of hydrogen bonding with oxygenated surface groups from the adsorbent. Conversely, the surface becomes less protonated or negatively charged at pH closer to or above pH_pzc_, weakening these interactions and thus reducing the adsorption capacity. It was concluded that the higher acidic functional group concentration (mainly carboxylic groups), microporosity, and surface area of activated carbon 3A compared to those of activated carbon 3B favored the interactions with MEK molecules via hydrogen bonding and polar surface interactions. 

**Figure 7:**
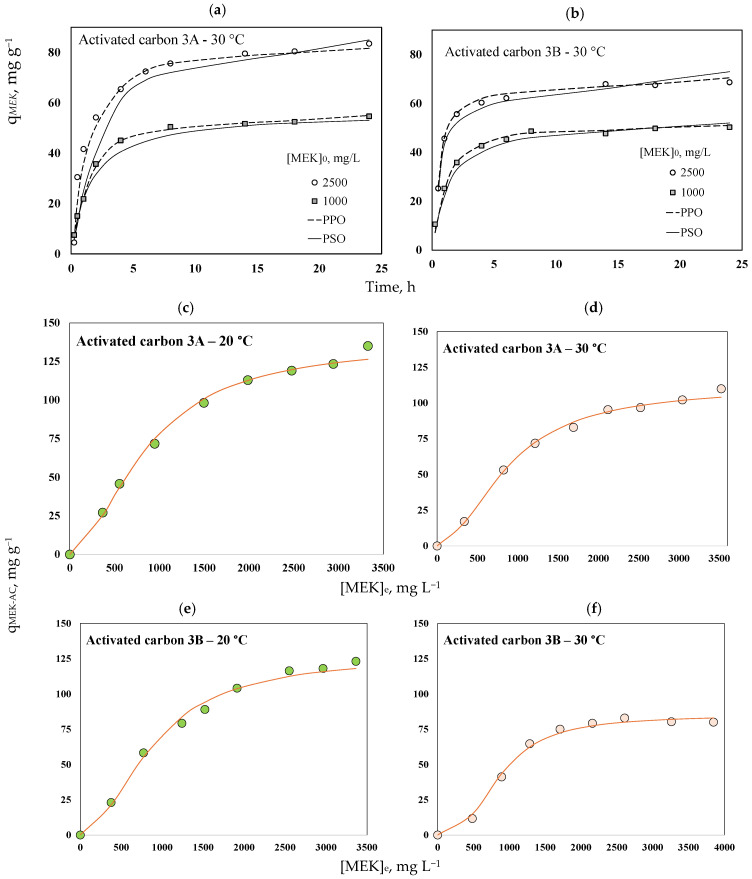
MEK adsorption data. (**a**,**b**) Kinetics using activated carbons 3A and 3B at pH 6. [MEK]_0_ = 1000 and 2500 mg L^−1^. Isotherms using activated carbons (**c**,**d**) 3A and (**e**,**f**) 3B at pH 6 and 20–30 °C, and their simulation with a double layer model.

Adsorption kinetics for MEK separation using these activated carbons are reported in Figure 7a,b. The separation of this organic molecule from aqueous solutions occurred quickly in the first hours, and the equilibrium was achieved at >10 h; 90% of the total MEK separation was completed at 8 h. This result was associated with the high surface area of both activated carbons [69]. It was found that the increase in initial MEK concentration favored the mass transfer and separation efficacy [81]. 

Kinetic parameters obtained for MEK separation are reported in Table 3. Calculated pseudo-first-order rates (k_1_) for MEK separation were 0.43–0.71 and 0.48–0.89 min^−1^ for activated carbons 3A and 3B, respectively, while pseudo-second-order rates (k_2_) were 0.003–0.011 and 0.007–0.012 g mg^−1^ h^−1^ for the same adsorbents. The pseudo-first-order model correlated better with the MEK separation kinetics (R^2^ = 0.985–0.998) for both activated carbons. This model indicated that the adsorption ratio was proportional to the number of binding sites available on the adsorbent surface [82,83]. The separation rate constant (k_1_) increased with increasing initial MEK concentration, demonstrating that mass transfer from the aqueous solution to the activated carbon surface was favored when more molecules of this organic compound were present in the fluid.

Adsorption isotherms for MEK separation are reported in Figure 7c–f. The S-type isotherm matched MEK equilibrium data of both activated carbons, suggesting a multilayer adsorption with the progressive adsorbent saturation [84,85]. The maximum experimental q_MEK-AC_ values of activated carbons 3A and 3B were 135 and 118 mg g^−1^, respectively, at 20 °C and pH 6. MEK separation decreased when the aqueous solution temperature increased to 30 °C, where the maximum experimental values of q_MEK-AC_ were 110 and 80 mg g^−1^ for these adsorbents, respectively. The reduction of MEK adsorption capacities was 19% and 32% for samples 3A and 3B, respectively, due to the temperature increase, indicating an exothermic separation with estimated ΔH° values of −7 and −13 kJ mol^−1^, respectively.

Similar results have been reported for the temperature effect on the adsorption-based separation of other organic compounds dissolved in aqueous solutions. Giraudet et al. [86] studied the adsorption of acetone, ethyl formate, and dichloromethane in water using commercial activated carbons at 20–80 °C. The results showed that high temperatures may enhance the adsorption capacities for the separation of acetone and ethyl formate. The authors indicated that this trend occurred because the increased kinetic energy enabled more volatile organic compound molecules to overcome energy barriers and interact with the adsorbent surface. In contrast, the dichloromethane adsorption capacity decreased because its chemical properties did not favor strong interactions with the adsorbent. Overall, it is expected that an increase in the temperature affects surface energy and molecular diffusion and reduces the adsorbate–adsorbent interaction energy due to the exothermic nature (i.e., ΔH° < 0) of the separation process. Higher kinetic energy of MEK molecules can lead to shorter contact time with active sites, which, combined with potential molecular desorption, explains the observed decreasing trend of adsorption capacity at 30 °C [87,88,89,90]. It is convenient to recall that absolute values of ΔH° < 20 kJ mol^−1^ indicate a physisorption-based mechanism [49]. 

The results of MEK adsorption isotherm modeling are presented in Table 3. Statistically, the best fit was obtained using the Sips model (R^2^ = 0.991–0.998), followed by the Langmuir (R^2^ = 0.758–0.954), and the Freundlich R^2^ = 0.713–0.912) isotherms. The Sips model combines the characteristics of the Langmuir isotherm (which assumes monolayer adsorption) and the Freundlich model (which considers the presence of heterogeneous systems, including multilayer adsorption). The data fitting results showed that parameter *n_S_* of the Sips model ranged from 0.4 to 0.8, suggesting the presence of a heterogeneous adsorption system with the potential formation of multilayers.

**Table 3:** Modeling results of MEK adsorption kinetics and isotherms using avocado seed-based activated carbon.

		Activated Carbon			
		3A		3B	
		[MEK]_0_, mg L^−1^			
Kinetic Model	Parameters	1000	2500	1000	2500
Pseudo-first order $q_{MEKt}=q_{MEKe}(1-\exp\left(-k_{1}t\right))$	k_1_, min^−1^	0.432	0.706	0.477	0.89
	q_MEKe_, mg g^−1^	49.30	81.16	63.54	80.31
	R^2^	0.993	0.985	0.998	0.995
Pseudo-second order $q_{MEKt}=\frac{q_{MEKe}^{2}\;k_{2}t}{1+q_{MEKe}\;k_{2}t}$	k_2_, g mg^−1^ h^−1^	0.011	0.003	0.007	0.012
	q_MEKe_, mg g^−1^	56.56	96.05	72.28	89.30
	R^2^	0.942	0.901	0.951	0.965
		**Separation Temperature, °C**			
**Isotherm Model**	**Parameters**	**20**	**30**	**20**	**30**
$q_{MEKe}=\frac{q_{MEKm}\;K_{L}\;{\left[MEK\right]}_{e}}{1+K_{L}\;{\left[MEK\right]}_{e}}$ Langmuir [91]	K_L_, L mg^−1^	3.9 × 10^−4^	3.2 × 10^−4^	2.9 × 10^−4^	1.0 × 10^−4^
	q_MEKm_, mg g^−1^	244.89	210.43	267.74	351.44
	R^2^	0.954	0.911	0.892	0.758
$q_{MEKe}=K_{F}\;{\left[MEK\right]}_{e}^{\frac{1}{n_{F}}}$ Freundlich [92]	K_F_, mg^1-/n^ L^1/n^ g^−1^	0.51	0.27	0.31	0.05
	n_F_	1.44	1.34	1.33	1.09
	R^2^	0.912	0.857	0.851	0.713
$q_{MEKe}=\frac{q_{MEKm}\;K_{s}\;{\left[MEK\right]}_{MEKe}^{n_{S}}}{1+K_{s}\;{\left[MEK\right]}_{e}^{n_{S}}}$ Sips [93]	K_s_, L^n^ (mg^n^)^−1^	6.5 × 10^−5^	1.3 × 10^−4^	3.3 × 10^−5^	1.1 × 10^−8^
	n_S_	0.73	0.83	0.68	0.37
	q_MEKm_, mg g^−1^	138.87	115.03	112.84	86.08
	R^2^	0.992	0.998	0.996	0.991

**Figure 8:**
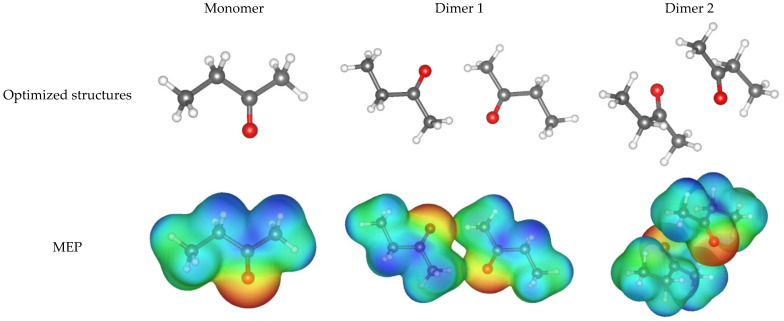
Optimized structures and molecular electrostatic potential (MEP) of monomer and dimers of MEK.

DFT simulations and statistical physics theory were applied to improve the analysis of the MEK adsorption mechanism. DFT calculations indicated that the formation of MEK molecular aggregates was feasible, see Figure 8. The two dimers were found to be thermodynamically stable and could be formed in an aqueous solution. Specifically, the interaction energies calculated via DFT were −16.7 and −30.2 kJ mol^−1^ for the dimers 1 and 2, respectively, which could occur in an aqueous solution. The molecular dimensions of MEK dimers and monomers obtained from the DFT simulations are reported in Table 4. DFT simulations indicated that dimer 2 was the most feasible MEK species to adsorb on the tested activated carbons. Therefore, a double-layer adsorption model with one interaction energy [91] was used to correlate the MEK adsorption isotherms. This model was defined as

(1) $$q_{MEK-AC}=n_{MEK}\;N_{MEK}\frac{{\left(\frac{{\left[MEK\right]}_{e}}{{\left[MEK\right]}_{hs}}\right)}^{n_{MEK}}+2{\left(\frac{{\left[MEK\right]}_{e}}{{\left[MEK\right]}_{hs}}\right)}^{{2n}_{MEK}}}{1+{\left(\frac{{\left[MEK\right]}_{e}}{{\left[MEK\right]}_{hs}}\right)}^{n_{MEK}}+2{\left(\frac{{\left[MEK\right]}_{e}}{{\left[MEK\right]}_{hs}}\right)}^{{2n}_{MEK}}}$$

(2) $$E_{MEK}=RTln\left(\frac{{\left[MEK\right]}_{Sol}}{{\left[MEK\right]}_{hs}}\right)$$

(3) $$q_{Sat-MEK-AC}=2n_{MEK}\;N_{MEK}$$

where [MEK]_e_ is the MEK equilibrium concentration (mg L^−1^) in the aqueous solution after the separation process, [MEK]_Sol_ is the MEK solubility (mg L^−1^) in water, [MEK]_hs_ is the half MEK concentration (mg L^−1^) for the activated carbon saturation, R is the universal gas constant, T is the MEK separation temperature in Kelvin, n_MEK_ is a parameter that estimates the number of MEK molecules adsorbed per activated carbon active site, N_MEK_ is the concentration of active sites (reported here in mmol g^−1^) from activated carbon that participated in MEK separation, E_MEK_ is the interaction energy (kJ mol^−1^) involved in MEK separation, and q_Sat-MEK-AC_ (mg g^−1^) is the MEK adsorption capacity of activated carbon at the saturation condition.

MEK isotherms were adjusted by this double-layer model (R^2^ ≥ 0.99), and the results are reported in Figure 7c–f. MEK multi-molecular adsorption was confirmed by the statistical physics modeling, where n_MEK_ ranged from 1.33 to 1.41 for activated carbon 3A and from 1.43 to 2.0 for activated carbon 3B at 20 and 30 °C, respectively. It was calculated that 59–67% of MEK molecules adsorbed on the active sites of activated carbon 3A corresponded to monomers, while the remaining 33–41% corresponded to MEK dimers. For the case of activated carbon 3B, 43% of MEK molecules could be adsorbed forming two layers on the adsorbent surface at 20 °C, while only dimers were separated from the aqueous solution at 30 °C. 

**Figure 9:**
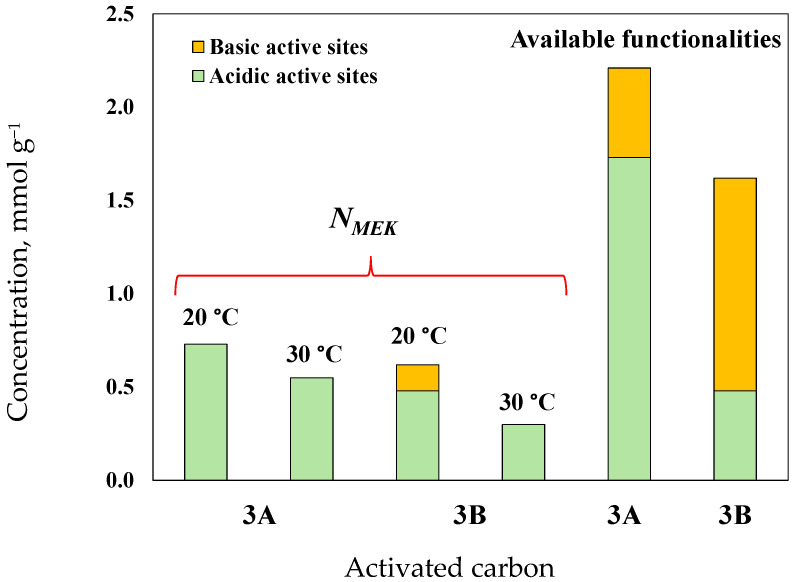
Concentration of active sites and calculated NMEK values for MEK separation from aqueous solutions using activated carbon samples 3A and 3B.

Calculated N_MEK_ values were 0.73 and 0.55 mmol g^−1^ for MEK adsorption on activated carbon 3A at 20 and 30 °C, respectively, and 0.62 and 0.30 mmol g^−1^ for activated carbon 3B at the same operating conditions. These results indicated that the concentration of active sites participating in MEK separation was reduced by 25% (3A) and 52% (3B) as the adsorption temperature increased. Therefore, it could be expected that MEK dimers were mainly adsorbed on the externally activated carbon surface at 30 °C because of steric restrictions on the adsorbent pores. In contrast, the MEK monomers were primarily adsorbed on the active sites located in the internal porous material structure. MEK adsorption on activated carbon 3A involved mainly acidic functional groups, while both basic and acidic sites of activated carbon 3B participated in the separation of this organic compound, see Figure 9. Calculated q_Sat-MEK-AC_ values of these adsorbents correlated with their surface areas, i.e., 113–140 mg g^−1^ (421 m^2^ g^−1^) for activated carbon 3A > and 85–129 mg g^−1^ (309 m^2^ g^−1^) for activated carbon 3B. However, their adsorption properties were not directly proportional to the improvement in adsorbent textural parameters caused by both chemical and thermal activations of avocado-based char, highlighting the importance of oxygenated surface functionalities in MEK separation. *E_MEK_* values of 11–12 kJ mol^−1^ were estimated for this adsorption process, which were consistent with the proposed MEK physisorption mechanism via hydrogen bonding and electrostatic interactions. 

In summary, the integrated analysis of the MEK separation mechanism indicated that the adsorption capacity increased with a decrease in the aqueous solution pH. Depending on the solution pH condition and adsorbent pH_pzc_, the oxygenated functional groups of these activated carbons can be deprotonated [47], and their surfaces can be positively or negatively charged, generating electrostatic interactions with MEK molecules. Activated carbon surface and MEK molecular structure contain oxygenated functional groups that could participate in MEK separation via hydrogen bonding [92,93]. Therefore, the physisorption mechanism for MEK separation from aqueous solutions using tested activated carbons involved non-covalent forces [93]. Preliminary studies indicated that MEK desorption from tested activated carbon samples was feasible, for example, when using methanol or ethanol. These results are also evidence of the physisorption mechanism to separate MEK from aqueous solutions. Further studies are required to optimize the regeneration conditions of the materials tested in this study.

Finally, a survey of MEK separation studies using various adsorbents under different operating conditions is reported in Table 5, where their q_MEK_ ranged from 15 to 470 mg g^−1^. For instance, it has been reported that a MEK separation from water can be achieved using a commercial granulated activated carbon with an adsorption capacity of 60 mg g^−1^ [15], 275 mg g^−1^ with organic clay beads [94], and 15 mg g^−1^ via a fixed-bed column packed with sepiolite [8]. These results confirmed that the avocado-based activated carbon prepared with H_2_SO_4_ can be an effective and competitive adsorbent, which is prepared using a circular economy approach, to separate MEK from fermentation broths.

## 3. Materials and Methods

### 3.1. Preparation and Tailoring of Avocado-Based Activated Carbons

**Figure 10:**
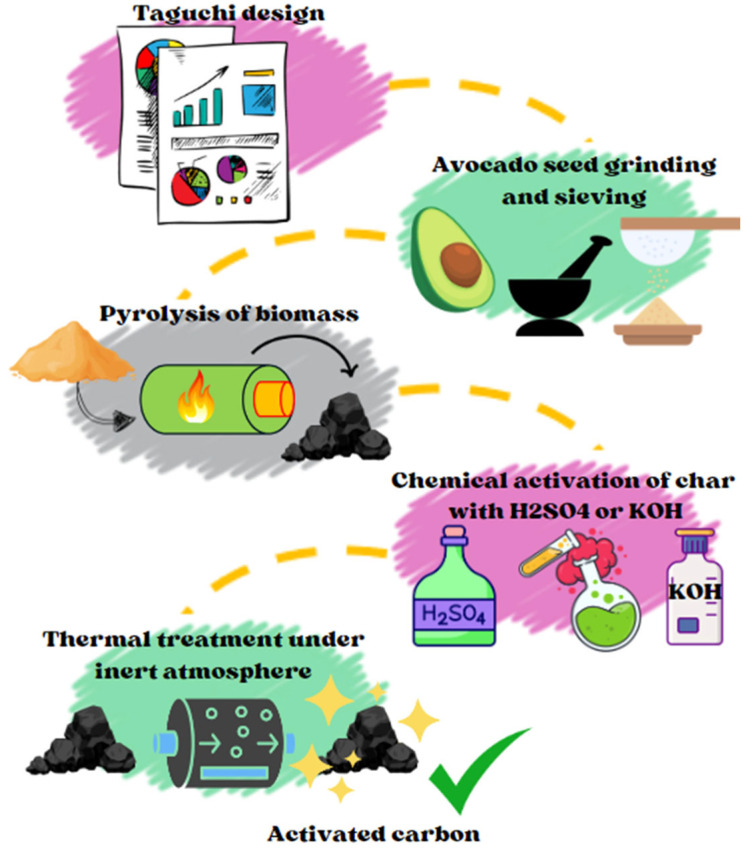
Synthesis route for the preparation of activated carbon samples from avocado seeds to separate MEK from aqueous solutions.

An orthogonal Taguchi L_9_ experimental design was applied to prepare 18 activated carbon samples using different synthesis conditions, activators (i.e., H_2_SO_4_ or KOH), and avocado seeds as the precursor. Table 6 provides the tested experimental conditions for obtaining the activated carbon samples, and Figure 10 illustrates the flowchart of their synthesis route. The residual biomass was ground (0.4–0.5 mm), washed, and dried at 50 °C for 48 h. Char samples were obtained via biomass pyrolysis using a tubular furnace (Carbolite Eurotherm, Carbolite Gero, Stuttgart, Germany) operated under an inert N_2_ atmosphere (400 mL min^−1^) and a heating ramp of 10 °C min^−1^ to reach the target pyrolysis temperatures. The avocado chars were activated using H_2_SO_4_ or KOH for 8 h at 30 °C under constant stirring. After chemical activation, the modified chars were separated from the suspension containing the activator and washed with deionized H_2_O to remove excess reagents. Thermal activation was applied to these samples, under an N_2_ atmosphere at 200 mL min^−1^, for 2 h at 500–900 °C to generate the final avocado-based activated carbons. The biomass pyrolysis time and temperature to obtain the avocado chars, the concentration of H_2_SO_4_ or KOH solutions used as chemical activators to modify the surface of the char samples, and the temperature of the final thermal activation to produce the avocado-based activated carbons were the main variables analyzed in this study. The results from the L_9_ experimental design allowed the analysis of preparation conditions and their impact on the surface properties of activated carbons and the identification of conditions that improved their MEK adsorption capacities. 

MEK separation using the activated carbon samples from Table 6 was assessed via batch adsorption experiments using 0.02 g of adsorbent and 10 mL of MEK solution with an initial concentration ([MEK]0) of 2000 mg L^−1^. The adsorbent dosage used in the experiments was optimized via preliminary tests. MEK separation was tested at pH 6 and 30 °C for 24 h under constant stirring at 120 rpm. For comparative purposes, additional MEK separation studies were performed using avocado chars without chemical activation and commercial bone char. The results of these adsorbents were the reference for the separation assessment. All separation tests were performed in triplicate, and the average values were reported and used for statistical data analysis.

The MEK adsorption capacity (q_MEK_, mg g^−1^) of the tested adsorbents was calculated using the mass balance from the results of the batch adsorbers, where MEK content in the aqueous solution was measured via gas chromatography following the conditions and procedure reported in the Supplementary Information.

The signal-to-noise (S/N) ratio analysis [95] was carried out using the experimental q_MEK_ values, where a ratio (Rq) of MEK adsorption capacities obtained from the avocado activated carbon (q_MEK-AC_) and its corresponding char without activation (q_MEK-C_) was determined. The following expressions were applied

(4) $$\frac{S}{N}=-10log\left(\frac{1}{3}\sum_{i=1}^{3}\left[\frac{1}{R_{q}^{2}}\right]\right)$$

(5) $$Rq=\frac{q_{MEK-AC}}{q_{MEK-C}}$$

where Rq > 1 indicates that the MEK adsorption capacity of avocado-activated carbon improved due to avocado char prepared under the same pyrolysis conditions. An analysis of variance (ANOVA) was carried out to identify the individual contribution of each variable studied in the activated carbon preparation and to establish the best pyrolysis and activation conditions for enhancing MEK separation from aqueous solutions. It is convenient to recall that q_MEK-AC_ and q_MEK-C_ were quantified under identical experimental adsorption conditions. The perspective “higher is better” was applied in the S/N analysis using the MINITAB^®^ 18 software.

### 3.2. Kinetics and Isotherms for MEK Separation Using Avocado-Based Activated Carbon

The best avocado activated carbon obtained from ANOVA and S/N analyses was used to characterize the thermodynamics of MEK separation from aqueous solutions. Pseudo-first and pseudo-second order rates [82,96,97] for MEK adsorption were calculated from experimental kinetic data obtained with [MEK]_0_ = 1000 and 2500 mg L^−1^ at pH 6 and 30 °C. MEK adsorption equilibrium was studied at pH 6 and 20–30 °C using [MEK]_0_ = 500–4000 mg L^−1^ and a contact time of 24 h. Langmuir [98], Freundlich [99], and Sips [100] models were used to correlate the experimental MEK adsorption isotherms for obtaining a preliminary overview of the adsorption mechanism. A statistical physics model [101] was proposed to complete the discussion of the MEK adsorption mechanism. The selection of this model was based on the surface characterization results and DFT calculations. The details of the DFT modeling are provided in the Supplementary Information. Statistica^®^ 13 software was used in the MEK adsorption data fitting. The enthalpy change (ΔH°, kJ mol^−1^) for MEK adsorption was estimated from the experimental equilibrium data at 20–30 °C following the methodology described by Tran et al. [102]. MEK separation was also analyzed by varying the solution pH from 4 to 7 using [MEK]_0_ = 3000 mg L^−1^ at 20 °C for 24 h. All the experiments were performed in duplicate using an adsorbent dosage of 2 g L^−1^ under continuous stirring at 120 rpm.

### 3.3. Surface Chemistry Characterization of Avocado-Based Char and Activated Carbon

Samples collected at different stages of the avocado-based activated carbon preparation route, with and without loaded MEK, were selected to analyze and explain the changes in their surface chemistry. The chemical compositions of the activated carbon samples were determined by X-ray fluorescence (XRF), and their crystalline structures were characterized by X-ray diffraction (XRD). The surface functional groups were identified using Fourier Transform Infrared Spectroscopy (FTIR). The specific surface area and pore volume of the activated carbon samples were estimated from the N_2_ isotherm data at 77 K using the BET and BJH models. The morphologies of the tested samples were observed by scanning electron microscopy (SEM). Acidic and basic sites and pH_pzc_ were determined using the procedure described by Faria et al. [103]. The details of these characterization techniques are provided in the Supplementary Information.

## 4. Conclusions

MEK separation from aqueous solutions was studied using activated carbons prepared from avocado seeds, as well as through chemical and thermal activations. H_2_SO_4_ activation was more effective than KOH activation in tailoring the surface properties of avocado-based activated carbon for MEK separation, where the pyrolysis temperature applied to obtain the avocado char was a key synthesis parameter. MEK separation using the best avocado-based activated carbon was an exothermic process and highly pH-dependent. DFT and statistical physics modeling results confirmed the presence of MEK molecular aggregates in the aqueous solutions. Hydrogen bonding and electrostatic interactions played a relevant role in the double-layer adsorption of this organic compound from aqueous solutions. Calculated saturation MEK adsorption capacities of 140 and 129 mg g^−1^ were obtained for the adsorbent samples activated with H_2_SO_4_ and KOH, respectively, at 20 °C and pH 4. The improvement in MEK adsorption properties of avocado-based activated carbon prepared with H_2_SO_4_ was associated with the incorporation of acidic functional groups, mainly carboxylic groups, and an increase in the adsorbent surface area. The best avocado-based activated carbon exhibited interesting adsorption properties, suggesting its potential for incorporation in downstream separation to recover MEK from fermentation broths and reduce its release as an environmental pollutant. The preparation of tailored adsorbents from avocado seeds can offer economic and sustainability benefits due to the abundance of this agro-industrial waste, in line with the principles of the circular economy. These factors position these materials as competitive and environmentally friendly alternatives to commercial products. It is recommended that future studies focus on the regeneration of these materials as a critical aspect, since multiple parameters are involved in assessing the MEK desorption and restoration of the adsorbent properties.

## Figures and Tables

**Table 1:** Pyrolysis yields and pH_pzc_ of avocado-based chars prepared under different pyrolysis conditions.

	Pyrolysis Conditions to Obtain Avocado Seed Char			
Char Sample	Temperature, °C	Dwell Time, h	Yield, %	pH_pzc_
1	700	2	26.20	6.76
2	700	4	25.37	6.75
3	700	6	25.12	6.78
4	800	2	24.84	6.79
5	800	4	24.52	6.77
6	800	6	23.35	6.85
7	900	2	24.27	6.84
8	900	4	23.05	6.94
9	900	6	22.25	7.19

**Table 2:** X-ray fluorescence elemental analysis of the avocado seed biomass and its char and activated carbons.

Adsorbent	Element, % (by Mass)						
	CHON	K	Ca	P	Fe	Mg	S
Avocado seed	97.12	2.59	1.23	0.37	ND	0.21	0.22
Char 3	96.51	ND	1.63	0.84	0.40	0.28	0.26
Activated carbon 3A	97.06	ND	1.06	0.87	ND	0.36	0.65
Activated carbon 3B	93.42	3.15	1.62	0.83	0.39	0.32	0.27

ND: Not detected.

**Table 4:** Calculated dimensions, volumes, and interaction energies of the optimized structures for MEK monomer and dimers.

Structure	Dimensions, Å			Volumes, Å^3^	Interaction Energy, kJ mol^−1^
	X	Y	Z		
MEK	5.6	3.1	1.7	83.9	-
Dimer 1	11.4	9.2	6.8	174.0	–16.7
Dimer 2	8.1	7.5	6.7	176.2	–30.2

**Table 5:** MEK adsorption by different adsorbents under several operational conditions.

Adsorbent	Operational Conditions	MEK Adsorption Capacity, mg g^−1^	Reference
Commercial granulated activated carbon	25 °C, 15 h, batch system	60	Sotelo et al. [15]
siliceous rocks	25 °C, pH 7, 0.1 g, batch systems	470	Ali et al. [17]
Poly(vinyl alcohol) (PVA)/peat/organoclay composite beads	1 g, pH 6, 35 °C, 35 h, [MEK]_0_ = 500 mg L^−1^, batch system	275	Chan et al. [94]
HNO_3_-modified Sepiolite with *Streptococcus equisimilis* biofilm	Packed-bed columns with 90 g, binary solutions containing MEK and DEK at 100 mg L^−1^ each one, flow of 1 mL min^−1^ for 120 h	15	Silva et al. [8]
Avocado seed pyrolyzed and modified with H_2_SO_4_	20 °C, pH 4, 24 h, [MEK]_0_ = 3000 mg L^−1^, 2 g L^−1^ of adsorbent	142	This study
Avocado seed pyrolyzed and modified with KOH		131	This study

**Table 6:** Preparation conditions of activated carbon samples from avocado seed residues for MEK separation from aqueous solutions.

	Pyrolysis Conditions to Obtain Avocado Char		Activation Conditions of Avocado Char	
Sample	Temperature, °C	Dwell Time, h	Activator Concentration, M	Thermal Activation Temperature, °C
1	700	2	0.25	500
2	700	4	0.5	700
3	700	6	1.0	900
4	800	2	0.5	900
5	800	4	1.0	500
6	800	6	0.25	700
7	900	2	1.0	700
8	900	4	0.25	900
9	900	6	0.5	500

## Data Availability

Data is available upon request.

## Supplementary Materials

The following supporting information can be downloaded at: https://www.mdpi.com/article/10.3390/molecules30163426/s1, (a) Calculation of adsorption capacities, (b) Characterization techniques, (c) DFT calculations, Table S1. ANOVA results of the Taguchi L9 experimental design used in the preparation of avocado-based activated carbons for the MEK separation from aqueous solutions, (d) References. Refs. [104,105,106,107,108] are cited in the Supplementary Materials.

## Abbreviations

The following abbreviations are used in this manuscript:

MEK	Methyl ethyl ketone
