# Supplementary material for: Separation of the Biofuel Methyl Ethyl Ketone from Aqueous Solutions Using Avocado-Based Activated Carbons: Synthesis Conditions and Multilayer Adsorption Properties
Source: Molecules. 2025 Aug 20;30(16):3426. doi: 10.3390/molecules30163426 (PMC12388736; doi:10.3390/molecules30163426)
Supplement: Supplementary file 1 [file molecules-30-03426-s001.zip › molecules-3778796-supplementary.pdf]

SUPPLEMENTARY INFORMATION

(A) Calculation of adsorption capacities

MEK adsorption capacities q_{MEK} of tested activated carbon samples were obtained from batch adsorption studies using the next expression

$$q_{MEK} = \frac{([MEK]_0 - [MEK]_f)V}{m} \quad \text{(S1)}$$

where $[MEK]_0$ and $[MEK]_f$ are the initial and final concentrations of MEK, respectively; V is the volume of the MEK solution; and m is the mass of the adsorbent used in the experiments.

MEK quantification was performed using a Thermo Scientific Trace 1300 gas chromatograph with FID detector and TG 5 SLIMS column (30 m x 0.25 mm x 0.25 μm) composed of 5% diphenyl and 95% dimethylarylene siloxane. The methodology used for quantification was as follows: injection temperature of 90 °C in split mode, detector temperature of 300 °C, and helium as carrier gas. The oven was set with 0.5 min at 40 ºC and heating ramp of 10 ºC/min until reaching 100 °C, then the heating ramp increased to 20 °C/min until reaching 200 °C that was maintained for 8 min. A linear calibration curve was used for MEK quantification.

(B) Characterization techniques

X-ray fluorescence (XRF) analysis was performed with an EPSILON 4 spectrometer from Malvern-Panalytical and plastic capsules and Mylar windows of 3.6 micron was used. X-ray diffraction (XRD) analyses were carried out using an Empyrean diffractometer from Malvern Panalytical. The device was operated at 45 kV and 40 mA with a Bragg-Brentano configuration and CuKα1 radiation (λ = 1.5406 Å). All samples were analyzed at a scanning speed of 147 s with a step size of 0.02 ° 2θ. Fourier Transform Infrared Spectroscopy (FTIR) characterization was performed with a Nicolet iS10 infrared spectrophotometer from Thermo Scientific. For the collection of the infrared spectra, KBr-adsorbent pellets were prepared and a wavenumber range of 4000 - 400 cm^{-1} with 32 scans were used. Textural parameters of adsorbent samples were obtained with a volumetric sorption analyzer ASAP 2020 (Micromeritics) with nitrogen at 77 K. Samples were degassed during 48 h and the BET and BJH methods were employed to estimate specific surface area and pore volume. Scanning Electronic Microscopy (SEM) was done using a TM3000 Hitachi instrument. The basic sites of activated carbon samples were estimated by mixing 0.08 g of the adsorbent with 0.025 M HCl (10 mL) and stirring for 48 h at 30 °C. The adsorbent was decanted, and the remanent aqueous phase was titrated with 0.025 M NaOH. The acidic sites were determined by this procedure using a 0.025 M NaOH solution for the experiments and 0.025 M HCl as the titration agent. The pH at point of zero charge (pH_{pzc}) was obtained using NaCl 0.1 M at several pH values (pH_0). Specifically, 0.03 g of each adsorbent were put in contact with 10 mL of NaCl solution at a specific pH and stirred for 48 h at 30 °C.

The mixture was separated, and the final pH of the remaining solution was measured. The difference (ΔpH) between the pH_0 and final pH of the solutions was obtained, then the plot of ΔpH versus pH_0 with the intersection point of the resulting curve (ΔpH = 0) provided the pH_{pzc}.

(C) DFT calculations

DFT calculations were carried out using ORCA 5.0 program [104,105] to assess the molecular structure of MEK monomer and its dimer (MEK-MEK). Initial calculations were conducted at the B3LYP/6-311G(d,p) level of theory within the Khon-Sham scheme. For the final geometric optimization, the B3LYP/def2-TZVP level was applied. Dispersion corrections were incorporated via the D3 Grimme scheme [106], with the Becke-Johnson damping function [107]. Counterpoise corrections were considered for basis set superposition error (BSSE) [108]. Molecular electrostatic potential (MEP) was calculated, and the molecular volumes and dimensions were estimated using the Conolly Surface approximation. Interaction energy (ΔE) was calculated using the next equation:

$$\Delta E_{int} = E_{complex} - \left(E_{monomer_1} + E_{monomer_2}\right) \qquad \text{(S2)}$$

where $E_{complex}$ is the calculated energy of the MEK-MEK dimers, and $E_{monomer_1}$ and $E_{monomer_2}$ represent the calculated energies of the individual MEK molecular structure.

Table S1. ANOVA results of the Taguchi L_9 experimental designs used in the preparation of avocado-based activated carbons for the MEK separation from aqueous solutions.

Activator	*Preparation conditions*	*SS*	*Variance*
H_2SO_4	Pyrolysis temperature, °C	114.40	57.20
	Pyrolysis time, h	15.65	7.83
	Activator concentration, M	12.33	6.17
	Thermal activation temperature, °C	105.00	52.50
KOH	Pyrolysis temperature, °C	476.02	238.01
	Pyrolysis time, h	23.10	11.55
	Activator concentration, M	56.57	28.28
	Thermal activation temperature, °C	2.09	1.04
